# Medicinal Uses, Phytochemistry and Pharmacological Properties of *Elaeodendron transvaalense*

**DOI:** 10.3390/nu11030545

**Published:** 2019-03-04

**Authors:** Alfred Maroyi, Sebua Silas Semenya

**Affiliations:** 1 Medicinal Plants and Economic Development (MPED) Research Centre, Department of Botany, University of Fort Hare, Private Bag X1314, Alice 5700, South Africa; 2 Technology Transfer Office, Research Administration and Development Department, University of Limpopo, Private Bag X1106, Sovenga 0727, South Africa; sebusemenya@gmail.com

**Keywords:** Celastraceae, *Elaeodendron transvaalense*, herbal medicine, southern Africa

## Abstract

*Elaeodendron transvaalense* is a plant species, which is in high demand as a herbal medicine in southern Africa. This study critically reviewed the medicinal applications, phytochemistry and pharmacological activities of *E. transvaalense*. The literature on medicinal applications, phytochemical, and pharmacological activities of *E. transvaalense*, was collected from multiple internet sources including Elsevier, Google Scholar, SciFinder, Web of Science, Pubmed, BMC, Science Direct, and Scopus. Complementary information was gathered from pre-electronic sources, such as books, book chapters, theses, scientific reports, and journal articles obtained from the University library. This study revealed that the species is used as herbal medicine in 62.5% of the countries where *E. transvaalense* is native in southern Africa. It is mainly used as herbal medicine for diarrhoea, menorrhagia, stomach aches, skin infections, inflammations, and rashes. Phytochemical compounds identified from the species, include flavonoids, peltogynoid, phenols, proanthocyanidins, tannin, and triterpenes. Ethnopharmacological research revealed that extracts and phytochemical constituents isolated from *E. transvaalense* have antibacterial, antifungal, anti-HIV, anti-inflammatory, antioxidant, antiplasmodial, anti-protozoan, anti-pyretic, hypoglycaemic, larvicidal, cytotoxicity, and mutagenic activities. *Elaeodendron transvalense* should to be subjected to detailed phytochemical, pharmacological, and toxicological evaluations aimed at correlating the medicinal uses of the species with the ethnopharmacological properties of the species.

## 1. Introduction

*Elaeodendron transvaalense* (Burtt Davy) R. H. Archer is a shrub or small to medium-sized tree belonging to the Celastraceae family. The species is commonly known as anthill saffron or bushveld saffron. The synonyms of *E. transvaalense* include *Cassine transvaalensis* (Burtt Davy) Codd, *Crocoxylon transvaalense* (Burtt Davy) N. Robson, *E. croceum* (Thunb.) DC. var. *heterophyllum* Loes., *Pseudocassine transvaalensis* (Burtt Davy) Bredell and *Salacia transvaalensis* Burtt Davy [1,2]. The species has been recorded in deciduous woodland, along streams, rocky hillsides, and termite mounds in Botswana, Angola, Namibia, Mozambique, Zimbabwe, South Africa, Zambia, and Swaziland [1,2]. *Elaeodendron transvaalense* is popular as a traditional medicine in southern Africa and in South Africa, Raimondo et al. [3] categorized the species as Near Threatened using the IUCN Red List of Categories and Criteria of threatened species. There is a steady decline in the wild population of *E. transvaalense* in South Africa, which is attributed to over-harvesting, destructive harvesting of the bark, marketing of the plant products, and land clearing for agricultural and urbanization purposes. *Elaeodendron transvaalensis* was identified by both rural and urban herbalists as one of 15 species that are becoming increasingly rare in the KwaZulu-Natal province in South Africa [4], and was ranked twelfth among the most frequently demanded medicinal species in the same province [5]. *Elaeodendron transvaalense* is sold in informal herbal medicine markets in five of the nine provinces (55.6%) in South Africa, that is, the Eastern Cape, KwaZulu Natal, Gauteng, Limpopo, and the Western Cape [4,5,6,7,8,9,10,11,12,13,14]. Research by Williams et al. [7] showed that *E. transvaalense* was available in 48% to 70% of herbal medicine informal markets in Johannesburg, Gauteng province, and about 11,155 kg to 27,771 kg of the species’ bark were traded per annum as a herbal medicine in 2001 in Gauteng province alone [15]. Due to the increasing demand for the species, *E. transvaalense* is managed in herbal medicine home gardens in the Limpopo and North West provinces in South Africa [16,17]. This study reviewed the medicinal applications, phytochemical, and pharmacological activities of *E. transvaalense*, based on its therapeutic potential as a herbal medicine in southern Africa. Therefore, the aim of this review was to provide a detailed appraisal of the existing knowledge and literature on the medicinal uses, phytochemistry, biological activities, and pharmacological properties of *E. transvaalense*, in an attempt to create a database of information that can be used in future research aimed at exploring the therapeutic potential of the species.

## 2. Medicinal Uses of *Elaeodendron transvaalense*

Medicinal uses of the species have been recorded in Botswana, Swaziland, Namibia, Zimbabwe, and South Africa, accounting for 62.5% of the countries where *E. transvaalense* is native. The bark and root macerate of *E. transvaalense* are used as herbal medicines against several diseases in southern Africa, see Table 1. *Elaeodendron transvaalense* is used as herbal concoction for diarrhoea in South Africa and Swaziland [10,18,19,20,21,22,23], menorrhagia in Botswana, South Africa, and Zimbabwe [20,24,25], stomach aches in South Africa and Swaziland [19,20,21,22,23,26,27,28,29], skin infections, inflammations and rashes in Namibia, South Africa, and Swaziland [19,20,30,31,32]. The roots or root bark of *E. transvaalense* are mixed with the roots of *Peltophorum africanum* Sond. As a herbal medicine for female infertility [33] or mixed with roots of *Ozoroa paniculosa* (Sond.) R. Fern. & A. Fern. as a herbal medicine for high blood pressure [25]. The roots of *E. transvaalense* are mixed with *Drimia elata* Jacq. bulb, roots of *Elephantorrhiza elephantina* (Burch.) Skeels and *Zanthoxylum capense* (Thunb.) Harv., bark of *Sclerocarya birrea* (A. Rich.) Hochst. and *Sarcostemma viminale* (L.) R. Br. twigs as herbal medicines for human immunodeficiency virus (HIV) opportunistic infections [34] and sexually transmitted infections (STIs) [35]. Bark and leaves of *E. transvaalense* are used as an ethnoveterinary medicine for diarrhoea and worms [36,37].

## 3. Phytochemical Constituents of *Elaeodendron transvaalense*

**Figure 1:**
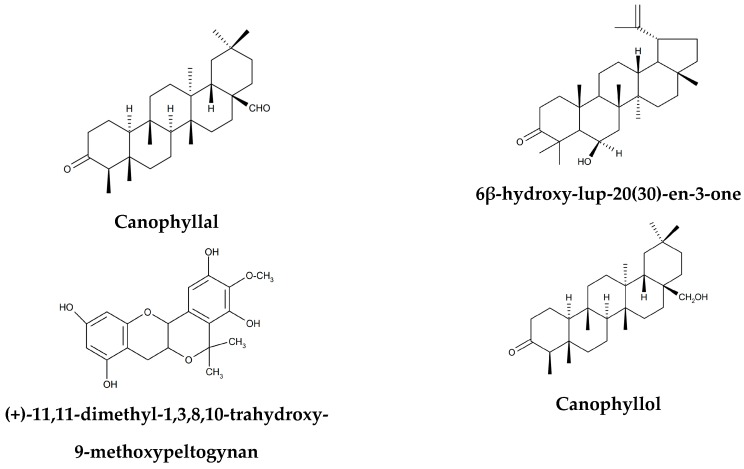

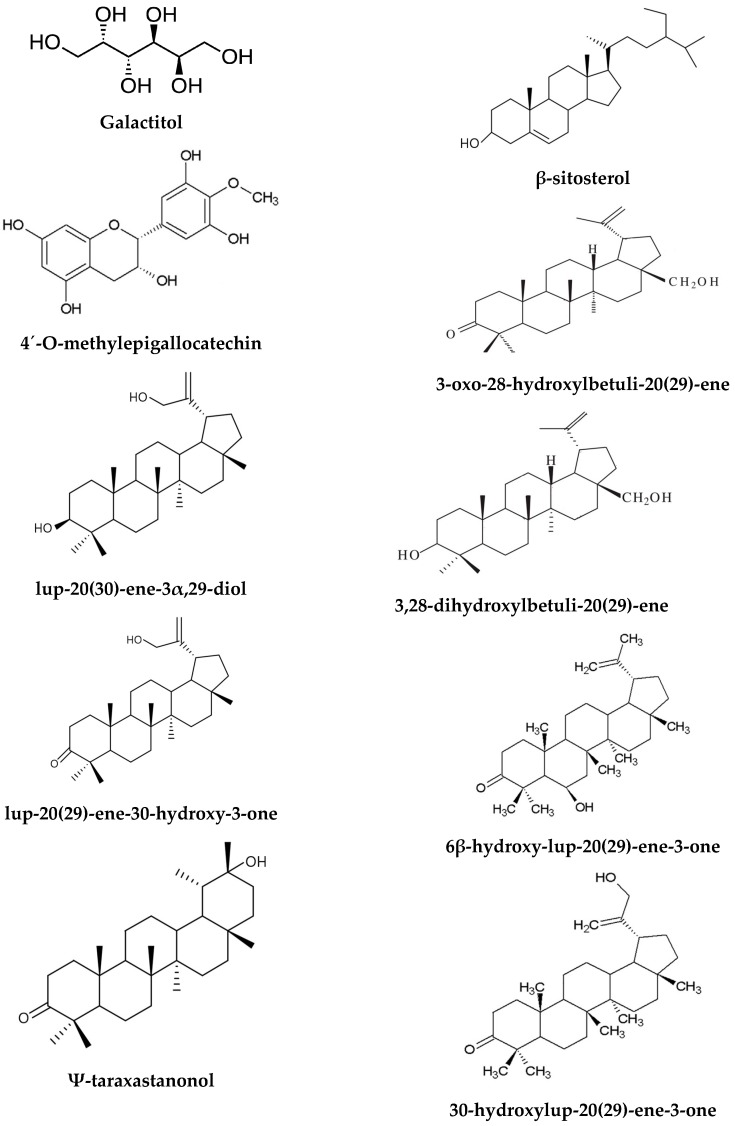
Chemical structures of compounds isolated from *Elaeodendron transvaalense*.

A wide range of minerals and phytochemicals (Table 2) have been isolated from the stem bark, leaves and the bark of *E. transvaalense*. Phytochemical screening of ethanol, hexane, and hexane: Ethyl acetate (80: 20) extracts of root and stem bark yielded carbohydrate, flavonoid, peltogynoid and triterpenes (Table 3; Figure 1). Drewes et al. [52] isolated canophyllal, (+)-11,11-dimethyl-1,3,8,10-trahydroxy-9-methoxypeltogynan, 6β-hydroxy-lup-20(30)-en-3-one, canophyllol and galactitol from the roots of *E. transvaalense*. Motlhanka et al. [39] isolated a flavonoid 4’-O-methyl-epigallocatechin from the aqueous root extract of *E. transvaalense*. Tshikalange and Hussein [53] isolated triterpenes lup-20(29)-ene-30-hydroxy-3-one, β-sitosterol, Ψ-taraxastanonol and lup-20(30)-ene-3α,29-diol and a flavonoid 4’-O-methyl-epigallocatechin from *E. transvaalense* bark ethanol extract. Mthethwa et al. [23] isolated triterpenoids, 3,28-dihydroxylbetuli-20(29)-ene and 3-oxo-28-hydroxylbetuli-20(29)-ene from the hexane: ethyl acetate (80: 20) bark extracts of *E. transvaalense*. Mamba et al. [46] isolated triterpenoids lup-20(30)-ene-3α,29-diol and lup-20(29)-ene-30-hydroxy-3-one, as well as a flavonoid 4’-O-methyl-epigallocatechin from *E. transvaalense* bark ethanol extract. Khumalo et al. [54] isolated triterpenes 30-hydroxylup-20(29)-ene-3-one, lup-20(30)-ene-3α,29-diol and 6β-hydroxy-lup-20(29)-ene-3-one and a flavonoid 4′-O-methyl-epigallocatechin from aqueous and dichloromethane stem bark extracts of *E. transvaalense*.

## 4. Pharmacological Activities

### 4.1. Antibacterial Activities

McGaw et al. [58] evaluated the antibacterial activities of aqueous, ethanol, and hexane bark extracts of *E. transvaalense* against *Bacillus subtilis, Escherichia coli, Klebsiella pneumoniae*, and *Staphylococcus aureus* using the disc-diffusion and micro-dilution assays, with neomycin (5 μg) as the positive control (Table 4). Ethanol and water extracts were active with minimum inhibitory concentration (MIC) values ranging from 0.1 mg/mL to 0.8 mg/mL against *Bacillus subtilis* and *Staphylococcus aureus* [58]. Samie et al. [59] evaluated the antibacterial activities of methanol root extracts of *E. transvaalense* against *Aeromonas hydrophila, Bacillus cereus, Bacillus pumilus, Bacillus subtilis, Enterobacter cloacae, Enterococcus faecalis, Escherichia coli, Klebsiella pneumoniae, Pantoea agglomerans, Proteus mirabilis, Pseudomonas aeruginosa, Salmonella cholerae-suis, Serratia marcescens, Shigella flexneri*, and *Staphylococcus aureus* using the disc diffusion and the microdilution methods with gentamicin as a positive control. The extracts showed activities against most of the tested microbes with the exception of *Klebsiella pneumoniae, Serratia marcescens*, and *Shigella flexneri* with the zone of inhibition ranging from 8 mm to 10 mm (Table 4). The extracts showed activities against *Aeromonas hydrophila, Bacillus pumilus, Bacillus subtilis, Enterobacter cloacae, Escherichia coli, Klebsiella pneumoniae, Proteus mirabilis, Salmonella cholerae-suis, Serratia marcescens*, and *Staphylococcus aureus*, with MIC values ranging from 6 mg/mL to >12 mg/mL [59]. Tshikalanga et al. [60] evaluated the antibacterial activities of aqueous and chloroform bark extracts of *E. transvaalense* against *Bacillus cereus, Bacillus pumilus, Bacillus subtilis, Enterobacter aerogenes, Enterobacter cloacae, Escherichia coli, Klebsiella pneumoniae, Pseudomonas aeruginosa, Serratia marcescens*, and *Staphylococcus aureus* using the agar dilution method. The extracts were active against *Bacillus cereus, Bacillus pumilus, Bacillus subtilis*, and *Staphylococcus aureus* with MIC values ranging from 20 mg/mL to 50 mg/mL [60]. Steenkamp et al. [31] evaluated the antibacterial activities of methanol and water bark extracts of *E. transvaalense* against *Escherichia coli*, *Staphylococcus aureus*, *Staphylococcus epidermidis*, and *Pseudomonas aeruginosa* using the plate-hole diffusion and broth microdilution methods with ampicillin as the positive control. The extracts were active against *Staphylococcus aureus* and *Staphylococcus epidermidis* exhibiting MIC values ranging from 1.3 mg/mL to 17.2 mg/mL and the positive control exhibited MIC value of 0.2 mg/mL [31]. Mthethwa et al. [23] evaluated antibacterial activities of *E. transvaalense* bark extracts against *Staphylococcus aureus* and *Staphylococcus epidermidis* using Kirby-Bauer disk diffusion and micro-dilution techniques with cloxacillin and dimethyl sulfoxide (DMSO) as positive, and negative controls, respectively. The extracts exhibited activities with zones of inhibition ranging from 23 mm to 31 mm (Table 4). The MIC values ranged from 0.6 µg/mL to 0.02 µg/mL [23]. Okem et al. [55] evaluated the antibacterial activities of ethanol stem bark extracts of *E. transvaalense* against *Escherichia coli* and *Staphylococcus aureus*, using the microdilution assay with neomycin as the positive control. The extracts exhibited activities with MIC values ranging from 0.8 mg/mL to 3.1 mg/mL [55]. Mamba et al. [46] evaluated the antibacterial activities of ethanol bark extracts of *E. transvaalense* and the compounds lup-20(30)-ene-3α,29-diol, lup-20(29)-ene-30-hydroxy-3-one and 4’-O-methyl-epigallocatechin isolated from the species against *Gardnerella vaginalis, Neisseria gonorrhoeae*, and *Oligella ureolytica* using the serial broth microdilution assay with ciprofloxacin as a positive control. The extracts and compounds exhibited activities with MIC values ranging from 1.6 mg/mL to 12.5 mg/mL, while the control exhibited MIC value of 0.01 mg/mL [46]. Khumalo et al. [54] evaluated antibacterial activities of dichloromethane and methanol stem bark extracts of *E. transvaalense* and compounds lup-20(30)-ene-3α,29-diol, 6β-hydroxy-lup-20(29)-ene-3-one, 30-hydroxylup-20(29)-ene-3-one and 4′-O-methylepigallocatechin isolated from the species against *Escherichia coli, Pseudomonas aeruginosa, Salmonella typhimurium, Shigella sonnei, Staphylococcus aureus*, and *Staphylococcus epidermidis* using the micro-titre plate broth two-fold serial dilution assay with ciprofloxacin as the positive control. The extract and the compounds demonstrated moderate antibacterial activities with MIC values ranging from 0.1 mg/mL to 1.7 mg/mL [54]. These findings corroborate the traditional use of the species as a herbal medicine for diarrhoea [10,18,19,20,21,22,23], sexually transmitted infections [16,35,45,46,50], skin infections [19,20,30,31,32], sore throat [32], stomach aches [19,20,21,22,23,26,27,28,29], venereal diseases [22,38], and wounds [51].

### 4.2. Anti-Fungal Activities

Steenkamp et al. [61] evaluated the anti-fungal activities of methanol and the water bark extracts of *E. transvaalense* against *Candida albicans* standard strain (ATCC 10231), and five clinical isolates using the plate-hole diffusion and broth microdilution methods, with amphotericin B as the positive control (Table 4). Only the methanol extract was active against the standard strain (ATCC 10231) exhibiting an MIC value of 20.2 mg/mL, while the positive control amphotericin B inhibited growth of all strains tested with an MIC value of <10 μg/mL [61]. Samie et al. [22] evaluated the anti-fungal activities of acetone and hexane bark extracts of *E. transvaalense* against *Candida albicans, Candida krusei*, and *Cryptococcus neoformans* using the agar diffusion and the microdilution methods, with nystatin and flucytosine as positive controls. Only hexane extract exhibited activities with the zone of inhibition ranging from 8 mm to 16 mm in comparison to 22 mm exhibited by both nystatin and flucytosine, the two positive controls. The MIC values against tested pathogens ranged from 0.5 mg/mL to 1.9 mg/mL, while the positive controls, nystatin and flucytosine, exhibited MIC values of 0.2 µg/mL, and 1.9 µg/mL, respectively. The minimum fungicidal concentration (MFC) values ranged from 1.9 mg/mL to 7.5 mg/mL (Table 4). The time-to-kill experiments indicated an intense time-dependent fungicidal effect of the hexane extract against *Candida albicans*, able to kill >90% of all the cells at a concentration of 1.9 mg/mL after a 10 hour incubation [22]. Mamba et al. [46] evaluated the antifungal activities of ethanol bark extracts of *E. transvaalense* and the compounds lup-20(30)-ene-3α,29-diol, lup-20(29)-ene-30-hydroxy-3-one and 4’-O-methyl-epigallocatechin, isolated from the species against *Candida albicans*, using the serial broth microdilution assay. The extracts and compounds exhibited activities with MIC values ranging from 3.1 mg/mL to <12.5 mg/mL [46]. These documented antifungal activities corroborate the use of the species as herbal medicine against candidiasis in South Africa [40], skin infections, and rashes [19,20,30,31,32].

### 4.3. Anti-HIV Activities

Morobe et al. [62] evaluated the anti-HIV activities of methanolic bark extracts of *E. transvaalense*, using the anti-HIV-1_iiiB_ assay (Table 4). The extract exhibited the ability to inhibit HIV-1_iiiB_ with half maximal effective concentration (EC_50_) values of 0.1 µg/mL and 0.2 µg/mL [62]. Bessong et al. [44] evaluated the anti-HIV activities of aqueous and methanol root extracts of *E. transvaalense* by assessing their inhibitory properties against HIV-1 reverse transcriptase (RT). The strongest inhibition was against the ribonuclease H (RNase H) activity of RT with methanol and aqueous extracts exhibiting half maximal inhibitory concentration (IC_50_) values of 30.0 µg/mL, and 31.2 µg/mL, respectively, while the inhibitory on RNA-dependent-DNA polymerase (RDDP) activity of RT for aqueous and methanol extracts exhibited IC_50_ values of 80.0 µg/mL, and 131.0 µg/mL, respectively [44]. Tshikalange et al. [50] evaluated the anti-HIV activities of 70% acetone, chloroform and ethyl acetate stem bark extracts of *E. transvaalense* by assessing their inhibition against α-glycohydrolase, reverse transcriptase, and viral proteins (NF-ĸB and Tat), which play a significant role in the HIV life cycle with mesuol as a positive control. In the in vitro assay of α-glycohydrolase, the extracts showed no inhibition against α-glycohydrolase, but the chloroform and ethyl acetate extracts showed good inhibitory activities of 64%, and 76%, respectively at the lowest concentration tested (1 µg/mL) in the NF-ĸB assay (Table 4). At the highest concentration 1 µg/mL, 70% acetone extract exhibited an inhibition of 54%, chloroform (73%) and ethyl acetate (75%), which was comparable to 84% exhibited by mesuol, the positive control. Chloroform and ethyl acetate extracts showed a high Tat inhibitory activity of 73%, and 75%, respectively at 15 µg/mL, while 70% acetone extract demonstrated a lower activity of 43%. The extracts showed lower cell death percentages, ranging from 17.1% to 27.6% after 36 h at the highest concentration tested (15 µg/mL) [50]. Mthethwa et al. [23] evaluated anti-HIV activities of *E. transvaalense* bark extracts using the anti-HIV-1_iiiB_ assay. The extract exhibited the ability to inhibit HIV-1_iiiB_ with half the maximal effective concentration (EC_50_) value of 3.5 µg/mL [23]. Mamba et al. [46] evaluated anti-HIV activities of ethanol bark extracts of *E. transvaalense* and the compounds lup-20(30)-ene-3α,29-diol, lup-20(29)-ene-30-hydroxy-3-one, and 4’-O-methyl-epigallocatechin isolated from the species against recombinant HIV-1 enzyme, using non-radioactive HIV-RT colorimetric assay with doxorubicin as a positive control. The ethanol extract exhibited low inhibitory activity of 20%, 4’-O-methyl-epigallocatechin showed moderate activity of 63.7%, while the positive control doxorubicin showed 96.5% inhibitory activity [46]. Sigidi et al. [63] evaluated the anti-HIV activities of aqueous bark extract of *E. transvaalense* using the reverse transcriptase (RT) assay. The extract showed inhibition ranging from 25% to 40% [63]. These documented anti-HIV activities corroborate the use of the species as herbal medicine against HIV opportunistic infections in South Africa [16,34,35,44].

### 4.4. Anti-Inflammatory Activities

Motlhanka and Habtemariam [64] evaluated the anti-inflammatory activities of aqueous crude root bark extract of *E. transvaalense*, using the cyclooxygenase (COX) inhibition assay, with indomethacin as a positive control (Table 4). The extract (125 mg/mL) exhibited 90% PGE_2_ inhibition in lipopolysaccharide (LPS) induced RAW 264.7 macrophages, which is comparable to 100% PGE_2_ inhibition exhibited by indomethacin, the control drug [64]. Mamba et al. [46] evaluated the anti-inflammatory activities of ethanol bark extracts of *E. transvaalense* and the compounds lup-20(30)-ene-3α,29-diol, lup-20(29)-ene-30-hydroxy-3-one, and 4’-O-methyl-epigallocatechin isolated from the species by assessing the inhibitory effects on the pro-inflammatory enzyme, 15-lipoxygenase (15-LOX), with quercetin as a positive control. The extracts and compounds exhibited activities with IC_50_ values, ranging from 31.4 µg/mL to 80.2 µg/mL, which was comparable to IC_50_ value of 48.9 µg/mL exhibited by quercetin, the control [46]. These findings support the traditional use of the species as herbal medicine for abdominal pains [24], body pains [20], skin inflammations [19,20,30,31,32], and wounds [51].

### 4.5. Antioxidant Activities

Motlhanka et al. [65] evaluated the antioxidant activities of water and ethanol root extracts of *E. transvaalensis* and a compound 4’-O-methyl-epigallocatechin, isolated from the species using the 2,2-dipheny-l-picrylhydrazyl (DPPH) free radical scavenging assay with quercetin, rutin, and ascorbic acid as positive controls. Above 100 µg/mL, the ethanolic extract showed an 80% scavenging activity, which was similar to the activities exhibited by the control antioxidant compounds quercetin, rutin, and ascorbic acid, and the water extract reached a similar of activity (80%) at 200 µg/mL (Table 4). Between 25.0 µg/mL to 50 µg/mL, the compound 4’-O-methyl-epigallocatechin exhibited a 65% scavenging activity, which was greater than the activities exhibited by both water and ethanol extracts. But at concentrations above 50 µg/mL, the scavenging activity of the ethanol extract exceeded that of the compound 4’-O-methyl-epigallocatechin [65]. Motlhanka et al. [39] evaluated the antioxidant activities of water and ethanol root extracts of *E. transvaalensis* and a compound 4’-O-methyl-epigallocatechin, isolated from the species, using the DPPH free radical scavenging assay with quercetin, rutin, and ascorbic acid as positive controls. Both the crude extract and the compound 4’-O-methyl-epigallocatechin showed activities, and at 100 µg/mL, the ethanolic extract showed 80% scavenging activity, which was similar to the activities exhibited by the control antioxidant compounds quercetin, rutin, and ascorbic acid; while the water extract reached a similar level at 100 µg/mL [39]. Nethengwe et al. [48] evaluated the antioxidant activities of methanolic bark extracts of *E. transvaalense*, using the DPPH free radical scavenging, 2,2’-azinobis-3-ethylbenzothiazoline-6-sulphonate (ABTS), hydroxyl (^•^OH) radical scavenging, super oxide (SO), nitric oxide (NO) radical scavenging, iron chelating property assays, total antioxidant capacity, and the sulphur hydryl (SH) content (Table 4). The IC_50_ values for the DPPH assay was 0.7 μg/mL, ABTS (4.1 μg/mL), iron chelating (3.9 μg/mL), ^•^OH (3.6 μg/mL), NO (3.6 μg/mL) and SO (1.6 μg/mL) [48]. Makhafola et al. [57] evaluated the antioxidant activities of methanolic leaf extracts of *E. transvaalense*, using the DPPH free radical scavenging assay with ascorbic acid as the positive control. The extract exhibited activities with EC_50_ value of 2.8 μg/mL, which was comparable to EC_50_ value of 2.3 μg/mL exhibited by ascorbic acid, the positive control [57]. The antioxidant activities exhibited by the crude extracts of *E. transvaalense* are probably due to flavonoids and phenolics, which have been isolated from the species [48,53,55,57].

### 4.6. Antiplasmodial Activities

Nethengwe et al. [48] evaluated the anti-plasmodial activities of aqueous, dichloromethane, and methanolic bark extracts of *E. transvaalense* against the chloroquine sensitive strain of *Plasmodium falciparum* (D10), using the parasite lactate dehydrogenase assay (Table 4). The other extracts were not active with the exception of dichloromethane, which exhibited IC_50_ value of 5.1 µg/mL [48]. These findings support the general view that *E. transvaalense* is a potential source of antimalarial agents and to some extent corroborate the traditional use of the species as herbal medicine against fever [10,20,21,23,26,29] and malaria [48].

### 4.7. Anti-Protozoan Activities

Fernandes et al. [66] evaluated the anti-protozoan activities of aqueous bark extract of *E. transvaalense* against *Trichomonas vaginalis*, using serial two-fold dilutions, with metronidazole as a positive control (Table 4). The extract showed activities with MIC value of 9.7 mg/mL while metronidazole exhibited MIC value of 0.5 µg/mL [66]. These findings corroborate the traditional use of the species as herbal medicine for sexually transmitted infections [16,35,45,46,50], skin infections [19,20,30,31,32], and venereal diseases [22,38].

### 4.8. Anti-pyretic Activities

Nethengwe et al. [48] evaluated the anti-pyretic activities of dichloromethane and methanolic bark extracts of *E. transvaalense*, using both female and male Sprague-Dawley rats with paracetamol as the reference drug (Table 4). The extracts exhibited the potential to reduce pyrexia in the induced rats and the activities were time- and concentration-dependent, with the extracts showing activity as early as 30 minutes, even at the lowest concentration of 100 mg/kg. The methanol extract showed significant activity that was comparable to paracetamol, the reference drug [48]. These findings corroborate the use of *E. transvaalense* as herbal medicine against fever [10,20,21,23,26,29].

### 4.9. Hypoglycaemic Activities

Deutschländer et al. [67] evaluated the hypoglycaemic activities of acetone stem bark extracts of *E. transvaalense*, by assessing their inhibiting effects on carbohydrate-hydrolising enzymes α-glucosidase and α-amylase. The acetone extracts were screened against C2C12 myocytes, 3T3-L1 preadipocytes and Chang liver cells by measuring their glucose uptake (Table 4). The in vitro assay in 3T3-L1 preadipocytes indicated that the extracts had potential of 138.6% to lower blood glucose levels at a concentration of 50 µg/mL. The α-glucosidase and α-amylase 50% inhibitory concentrations (IC_50_) of the extracts was found to be 50.6 µg/mL, and 1.1 µg/mL, respectively [67]. These results somehow support the usage of *E. transvaalense* as a herbal medicine against diabetes [66].

### 4.10. Larvicidal Activities

Nethengwe et al. [48] evaluated larvicidal activities of aqueous, dichloromethane, and methanolic bark extracts of *E. transvaalense*, using the mosquito larvicidal assay by the use of *Culex quinquefascitus* larvae. The results of the percentage mortality of the fourth instar larvae of *Culex quinquefascitus* showed that the aqueous extracts had least larvicidal activity of 35%, methanol (47%) and dichloromethane (60%) (Table 4). The IC_50_ values of methanol and dichloromethane extracts were 9.8 µg/mL and 18.2 µg/mL, respectively [48]. These findings corroborate the use of *E. transvaalense* as herbal medicine against malaria [48].

### 4.11. Cytotoxicity and Mutagenic Activities

Deutschländer et al. [67] evaluated the cytotoxic activities of stem bark extracts of *E. transvaalense*, by assessing its effects on preadipocytes and hepatocytes cell lines (Table 4). The extract exhibited cytotoxicity at 12.5 µg/mL to 3T3-L1 preadipocytes, and Chang liver cells [67]. Tshikalange and Hussein [53] evaluated the cytotoxicity activities of the crude ethanol extract and compounds lup-20(30)-ene-3α,29-diol, lup-20(29)-ene-30-hydroxy-3-one, Ψ-taraxastanonol, β-sitosterol, and 4’-O-methyl-epigallocatechin isolated from *E. transvaalense* bark extract, using the XTT (sodium 3’-[1-(phenyl amino-carbonyl)-3,4-tetrazolium]-bis-[4-methoxy-6-nitro] benzene sulfonic acid hydrate) colorimetric assay against Vero and MCF-7 breast cancer cell lines, with doxorubicin and zelaralenone as positive controls. The cell lines were inhibited by all the compounds at the highest concentration tested (200 µg/mL), with the exception of crude extract and Ψ-taraxastanonol. The crude extract, Ψ-taraxastanonol and 4’-O-methyl-epigallocatechin had little or no toxicity on Vero cells by exhibiting IC_50_ values greater than 100 µg/mL, while the crude extract and Ψ-taraxastanonol also exhibited IC_50_ values greater than 100 µg/mL in MCF-7 cell line. The IC_50_ values of other compounds in both Vero cells and MCF-7 cell line ranged from 19.4 µg/mL to 96.0 µg/mL [53]. Morobe et al. [62] evaluated the cytotoxic activities of methanolic and aqueous extracts of *E. transvaalense* against MAGI CCR5+ cells, using 3-(4,5-dimethylthiazol-2-yl)-2,5-diphenyl tetrazolium bromide (MTT) assay. The extracts exhibited activities with half maximal cytotoxic concentration (CC_50_) value of 3.7 mg/mL [62]. Nethengwe et al. [48] evaluated the cytotoxic activities of aqueous, dichloromethane, and methanolic bark extracts of *E. transvaalense*, using the MTT cell proliferation assay against human embryonic kidney (HEK293) and human hepatocellular carcinoma (HepG2) cells. The other extracts were not active with the exception of dichloromethane, which exhibited the median lethal concentration (LC_50_) value of 512.0 µg/mL and 394.0 µg/mL against HEK293, and HepG2, respectively [48]. Mthethwa et al. [23] evaluated the cytotoxic activities of *E. transvaalense* bark extracts, using the MTT assay with berberine as a positive control. The CC_50_ value of the extract was 200.0 µg/mL, which was higher than 27 µg/mL exhibited by berberine, the control and a selective index (SI) value of 57.1 [23]. Sigidi et al. [63] evaluated the cytotoxicity activities of aqueous bark extract of *E. transvaalense* on U937, MeWo, and Vero cell lines, using the MTT cell proliferation assay. The extract exhibited activities in all the three human tumour cancer cell lines [63].

Makhafola et al. [57] evaluated mutagenicity activities of methanolic leaf extracts of *E. transvaalense*, using the Ames test on *Salmonella typhimurium* strains TA98 and TA100. The authors also evaluated the antimutagenicity of the plant extracts against 4-nitroquinoline 1-oxide (4-NQO) using the Ames test. The extract did not exhibit any mutagenic activities, but showed weak antimutagenic activities (Table 4). The percentage inhibition of 4-NQO was 23.2% in *Salmonella typhimurium* TA98 and 21.3% in strain TA100 at the assayed concentration of 5 mg/mL [57].

## 5. Conclusion

The present review summarizes the medicinal uses, phytochemistry, and pharmacological properties *E. transvaalense*. The diverse pharmacological activities of *E. transvaalense* are somehow directly or indirectly involved in a range of physiological processes, which offer protection against both free radicals and harmful pathogens. In the past 30 years, *E. transvaalense* has been the subject of phytochemical and pharmacological research, but there is not yet enough data correlating the medicinal uses of the species with its phytochemical and pharmacological properties. Detailed studies on the pharmacokinetics, in vivo, and clinical research involving compounds isolated from *E. transvaalense* and extracts of the species are required. Therefore, future research should focus on the molecular modes or mechanisms of action, pharmacokinetics, and physiological pathways for specific extracts of the species, including the identification of the bioactive compounds of the species and their associated pharmacological activities. These studies need to be complemented with experimental animal studies, randomized clinical trials, and target-organ toxicity studies. The bark of *E. transvaalense* is known to be poisonous and there is need to do detailed toxicological evaluations that strike a balance between the medicinal potential, and adverse and toxic effects on the species. There is very little information on the toxicological properties of *E. transvaalense*, whether it causes superficial discomfort when ingested as herbal medicine or serious poisoning. In the absence of such detailed toxicological evaluations, the intake of *E. transvaalense* as a herbal medicine should, therefore, be done with caution as the species has potential to cause long-term damage in patients. The wide usage of *E. transvaalense* as a herbal medicine in southern Africa has resulted in an increased collection of its bark from the wild. The species population is declining due to harvesting for the medicinal plant trade, and this calls for conservation strategies and mechanisms to ensure sustainable utilization of the species.

## Figures and Tables

**Table 1:** Medicinal uses of *Elaeodendron transvaalense.*

Medicinal Use	Parts of the Plant Used	Country	References
Abdominal pains	Bark and roots	Zimbabwe	[24]
Anthelmintic	Root bark	South Africa	[22,38]
Arthritis	Root bark	Botswana	[39]
Backache	Root bark	Botswana	[25,39]
Bladder infections	Bark	South Africa	[22]
Blood cleanser	Roots	South Africa	[29]
Body pains	Bark	South Africa	[20]
Candidiasis	Roots	South Africa	[40]
Chest pains	Roots mixed with bulb of *Drimia elata* Jacq.	South Africa	[41]
Cough	Bark	South Africa	[22]
Diabetes	Bark	South Africa	[21]
Diarrhoea	Bark	South Africa and Swaziland	[10,18,19,20,21,22,23]
Emetic	Stem	Swaziland	[19,27]
Female infertility	Bark or roots mixed with *Peltophorum africanum* Sond. bark and decoction taken orally	South Africa	[33]
Fever	Bark	South Africa	[10,20,21,23,26,29]
Haemorrhoids	Root bark	South Africa	[22,38]
High blood pressure	Root bark mixed with roots of *Ozoroa paniculosa* (Sond.) R. Fern. & A. Fern.	Botswana	[42]
High blood pressure	Roots	Botswana	[43]
HIV/AIDS	Roots	South Africa	[16,44,45]
HIV/AIDS	Roots mixed with *Drimia elata* Jacq. bulb, roots of *Elephantorrhiza elephantina* (Burch.) Skeels and *Zanthoxylum capense* (Thunb.) Harv., bark of *Sclerocarya birrea* (A. Rich.) Hochst. and *Sarcostemma viminale* (L.) R. Br. twigs	South Africa	[34]
Induce vomiting	Stem bark	South Africa	[46,47]
Intestinal cramps	Bark	South Africa	[10,18,20,21]
Kidney infections	Bark	South Africa	[22]
Laxative	Bark	South Africa	[22]
Malaria	Bark	South Africa	[48]
Menorrhagia	Root bark	Botswana, South Africa and Zimbabwe	[20,24,25,49]
Menstrual problems	Root bark mixed with roots of *Ozoroa paniculosa*	Botswana	[42]
Sexually transmitted infections (STIs)	Roots and stem bark	South Africa	[16,45,46,50]
Sexually transmitted infections (STIs)	Roots mixed with *Drimia elata* bulb, roots of *Elephantorrhiza elephantina* and *Zanthoxylum capense*, bark of *Sclerocarya birrea* and *Sarcostemma viminale* twigs	South Africa	[35]
Skin infections, inflammations and rashes	Bark	Namibia, South Africa and Swaziland	[19,20,30,31,32]
Sore throat	Leaves	South Africa	[32]
Stomach aches	Bark and roots	Swaziland and South Africa	[19,20,21,22,23,26,27,28,29]
Stomach cleanser	Bark	South Africa	[10]
Venereal diseases	Root bark	South Africa	[22,38]
Wounds	Bark	Namibia	[51]
**Ethnoveterinary Medicine**			
Diarrhoea	Bark and leaves	South Africa	[36,37]
Worms	Leaves	South Africa	[37]

**Table 2:** Mineral and phytochemical composition of *Elaeodendron transvaalense*.

Mineral and Phytochemical Composition	Values	Plant Parts	References
Al (mg/kg dry weight (dw)	26.5–41.6	Stem bark	[55]
As (mg/kg dw)	0.06	Stem bark	[55]
Cr (mg/kg dw)	4.8	Stem bark	[55]
Cu (mg/kg dw)	2.8–3.5	Stem bark	[55]
Fe (mg/kg dw)	59.0–206.0	Stem bark	[55]
Flavonoid (mg/catechin equivalents/g dw)	0.1–0.2	Stem bark	[48,55]
Hg (mg/kg dw)	2.4–8.2	Stem bark	[55]
Mn (mg/kg dw)	11.3–12.7	Stem bark	[55]
Ni (mg/kg dw)	1.8–2.6	Stem bark	[55]
Pb (mg/kg dw)	1.2	Stem bark	[55]
Proanthocyanidin (mg/g)	0.25	Bark	[48]
Sn (mg/kg dw)	40.2–42.1	Stem bark	[55]
Sulphur hydryl (µg/g)	0.36	Bark	[48]
Tannin (mg/mL gallic acid equivalent)	0.4–0.8	Leaves	[56]
Total phenolics (mg of gallic acid equivalent/g of extract)	0.04–9.4	Bark and leaves	[48,55,57]
Zn (mg/kg dw)	3.8–4.4	Stem bark	[55]

**Table 3:** Phytochemical composition of *Elaeodendron transvaalense*.

Phytochemical Compound	Extract	Plant Part	References
**Carbohydrate**			
Galactitol	Ethanol	Root bark	[52]
**Flavonoid**			
4’-O-methyl-epigallocatechin	Dichloromethane, ethanol and water	Stem bark	[39,53,54]
**Peltogynoid**			
(+)-11,11-dimethyl-1,3,8,10-trahydroxy-9-methoxypeltogynan	Ethanol	Root bark	[52]
**Triterpenes**			
3-oxo-28-hydroxylbetuli-20(29)-ene	Hexane: ethyl acetate	Bark	[23]
3,28-dihydroxylbetuli-20(29)-ene	Hexane: ethyl acetate	Bark	[23]
30-hydroxylup-20(29)-ene-3-one	Dichloromethane and water	Root bark	[54]
6β-hydroxylup-20(29)-ene-3-one	Dichloromethane and water	Root bark	[54]
6β-hydroxy-lup-20(30)-en-3-one	Ethanol and hexane	Root bark	[52]
Canophyllal	Ethanol and hexane	Root bark	[52]
Canophyllol	Ethanol and hexane	Root bark	[52]
Lup-20(30)-ene-3α,29-diol	Dichloromethane, ethanol and water	Stem bark	[46,53,54]
Lup-20(29)-ene-30-hydroxy-3-one	Ethanol	Stem bark	[46,53]
β-sitosterol	Ethanol	Stem bark	[53]
Ψ-taraxastanonol	Ethanol	Stem bark	[53]

**Table 4:** Summary of pharmacological activities of *Elaeodendron transvaalense* crude extracts.

Activity Tested	Extract	Plant Part	Model	Effect	Reference
Antibacterial	Methanol	Bark	Kirby-bauer disk diffusion	Exhibited activities with zone of inhibition of 23 mm and 25 mm to 31 mm against *Staphylococcus aureus* and *Staphylococcus epidermis*, respectively	[23]
Antibacterial	Methanol	Bark	Micro-dilution technique	Minimum inhibitory concentration (MIC) values varied between 0.6 μg/mL and 0.02 μg/mL and extracts inhibited 6% of *Staphylococcus aureus* and 2% *Staphylococcus epidermidis* at a minimum concentration of 0.02 μg/mL	[23]
Antibacterial	Aqeous	Bark	Plate-hole diffusion and broth microdilution methods	Extracts exhibited activities with MIC values of 17.2 mg/mL against both *Staphylococcus epidermidis* and *Staphylococcus aureus*	[31]
Antibacterial	Methanol	Bark	Plate-hole diffusion and broth microdilution methods	Extracts exhibited activities with MIC values of 1.3 mg/mL and 2.5 mg/mL against *Staphylococcus epidermidis* and *Staphylococcus aureus*, respectively	[31]
Antibacterial	Ethanol	Bark	Serial broth microdilution	Extracts exhibited activities with MIC values of 12.5 mg/mL, 1.6 mg/mL and 3.1 mg/mL against *Gardnerella vaginalis, Neisseria gonorrhoeae* and *Oligella ureolytica*	[46]
Antibacterial	Dichloromethan	Bark	Micro-titre plate broth two-fold serial dilution assay	Extracts exhibited activities with MIC values of 0.4 mg/mL against *Pseudomonas aeruginosa*, 0.5 mg/mL against *Staphylococcus aureus* and *Staphylococcus epidermidis*, *Escherichia coli*, (0.7 mg/mL)*, Shigella sonnei* (0.8 mg/mL) and *Salmonella typhimurium* (1.0 mg/mL)	[54]
Antibacterial	Methanol	Bark	Micro-titre plate broth two-fold serial dilution assay	Extracts exhibited activities with MIC value of 1.3 mg/mL against *Escherichia coli, Staphylococcus aureus* and *Salmonella typhimurium*, 1.0 mg/mL against *Pseudomonas aeruginosa* and *Shigella sonnei*, and *Staphylococcus epidermidis* (1.7 mg/mL)	[54]
Antibacterial	Ethanol	Stem bark	Microdilution assay	Extracts exhibited activities with MIC values of 3.1 mg/mL and 0.78 to 1.6 mg/mL against *Escherichia coli* and *Staphylococcus aureus*, respectively	[55]
Antibacterial	Aqueous	Bark	Microdilution assay	Extracts exhibited activities with MIC values of 0.8 mg/mL and 0.2 mg/mL against *Bacillus subtilis* and *Staphylococcus aureus*, respectively	[58]
Antibacterial	Ethanol	Bark	Microdilution assay	Extracts exhibited activities with MIC values of 0.2 mg/mL and 0.1 mg/mL against *Bacillus subtilis* and *Staphylococcus aureus*, respectively	[58]
Antibacterial	Aqueous	Bark	Disc-diffusion assays	Extracts exhibited activities with MIC values of 0.2 mg/mL and 0.3 mg/mL against *Bacillus subtilis* and *Staphylococcus aureus*, respectively	[58]
Antibacterial	Ethanol	Bark	Disc-diffusion assays	Extracts exhibited activities with MIC values of 0.2 mg/mL and 0.6 mg/mL against *Bacillus subtilis* and *Staphylococcus aureus*, respectively	[58]
Antibacterial	Methanol	Roots	Disc diffusion method	Exhibited activities with zone of inhibition of 23 mm against *Bacillus cereus*, 8 mm against *Bacillus pumilus*, *Staphylococcus aureus, Enterococcus cloacae, Escherichia coli, Aeromonas hydrophila, Proteus mirabilis* and *Salmonella cholera-suis* and 10 mm against *Bacillus subtilis*, *Enterococcus faecalis*, *Pantoea agglomerans* and *Pseudomonas aeruginosa*	[59]
Antibacterial	Methanol	Roots	Microdilution method	Exhibited activities with MIC values of 12 mg/mL against *Bacillus pumilus, Bacillus subtilis, Enterococcus cloacae* and *Escherichia coli*, 6 mg/mL against *Klebsiella pneumoniae*, *Staphylococcus aureus* and *Salmonella cholera-suis* >12 mg/mL against *Aeromonas hydrophila, Proteus mirabilis* and *Serratia marcescens*	[59]
Antibacterial	Aqueous	Bark	Agar dilution method	Extracts exhibited activities with MIC values of 50.0 mg/mL against *Bacillus cereus* and *Bacillus pumilus*, 20.0 mg/mL against *Bacillus subtilis* and *Staphylococcus aureus*	[60]
Antifungal	Methanol	Bark	Plate-hole diffusion and broth microdilution methods	Extract exhibited activities with MIC value of 20.2 mg/mL	[61]
Antifungal	Hexane	Bark	Agar diffusion assay	Exhibited activities with zone of inhibition of 12 mm to 16 mm against *Candida albicans, Candida krusei* (8 mm to 14 mm) and *Cryptococcus neoformans* (14 mm to 16 mm)	[22]
Antifungal	Hexane	Bark	Microdilution assay	Exhibited activities with MIC values of 0.5 mg/mL against *Candida albicans* and 1.9 mg/mL against both *Candida krusei* and *Cryptococcus neoformans*	[22]
Antifungal	Hexane	Bark	Microdilution assay	Exhibited activities with minimum fungicidal concentration (MFC) values of 3.8 mg/mL against *Candida albicans, Candida krusei* (7.5 mg/mL) and *Cryptococcus neoformans* (1.9 mg/mL)	[22]
Antifungal	Hexane	Bark	Time-to-kill experiments	Extract was able to kill >90% of all cells of *Candida albicans* at a concentration of 1.9 mg/mL after a 10 hour incubation	[22]
Antifungal	Ethanol	Bark	Serial broth microdilution	Extracts exhibited activities with MIC values of 3.1 mg/mL against *Candida albicans*	[46]
Anti-HIV	Aqueous	Root	RNA-dependent-DNA polymerase (RDDP) activity of HIV-1 reverse transcriptase	Extracts exhibited activities with half maximal inhibitory concentration (IC_50_) value of 80.0 µg/mL	[44]
Anti-HIV	Methanol	Root	RNA-dependent-DNA polymerase (RDDP) activity of HIV-1 reverse transcriptase	Extracts exhibited activities with IC_50_ value of 131.0 µg/mL	[44]
Anti-HIV	Aqueous	Root	RNase H assay	Extracts exhibited activities with IC_50_ value of 31.2 µg/mL	[44]
Anti-HIV	Methanol	Root	RNase H assay	Extracts exhibited activities with IC_50_ value of 30.0 µg/mL	[44]
Anti-HIV	70% acetone	Stem bark	NF-ĸB assay	Extracts showed inhibitory activities of 45% to 54%	[50]
Anti-HIV	Chloroform	Stem bark	NF-ĸB assay	Extracts showed inhibitory activities of 57% to 73%	[50]
Anti-HIV	Ethyl acetate	Stem bark	NF-ĸB assay	Extracts showed inhibitory activities of 72% to 76%	[50]
Anti-HIV	70% acetone	Stem bark	HeLa-Tat-Luc assay	Extracts showed inhibitory activities of 22% to 43%	[50]
Anti-HIV	Chloroform	Stem bark	HeLa-Tat-Luc assay	Extracts showed inhibitory activities of 28% to 76%	[50]
Anti-HIV	Chloroform	Stem bark	HeLa-Tat-Luc assay	Extracts showed inhibitory activities of 63% to 75%	[50]
Anti-HIV	Methanol	Bark	Anti-HIV-1_iiiB_ assay	Exhibited activities with half maximal effective concentration (EC_50_) value of 0.1µg/mL and 0.2µg/mL	[62]
Anti-HIV	Methanol	Bark	Anti-HIV-1_iiiB_ assay	Exhibited activities with EC_50_ value of 3.5 µg/mL	[23]
Anti-HIV	Ethanol	Bark	HIV-RT colorimetric assay	Extract exhibited inhibitory activity of 20%	[46]
Anti-HIV	Aqueous	Bark	Reverse transcriptase (RT) assay	Extract showed inhibition ranging from 25% to 40%	[63]
Anti-inflammatory	Aqueous	Root bark	Cyclooxygenase (COX) inhibition assay	Extract exhibited 90% PGE_2_ inhibition in lipopolysaccharide (LPS) induced RAW 264.7 macrophages	[64]
Anti-inflammatory	Ethanol	Bark	Lipoxygenase (15-LOX) inhibitory assay	Extract exhibited activities with IC_50_ value of 80.2 µg/mL	[46]
Antioxidant	Aqueous	Roots	2,2-dipheny-l-picrylhydrazyl (DPPH) free radical scavenging assay	Above 200 µg/mL, the extract showed 80% scavenging activity	[65]
Antioxidant	Ethanol	Roots	DPPH free radical scavenging assay	Above 100 µg/mL, the extract showed 80% scavenging activity	[65]
Antioxidant	Aqueous	Roots	DPPH free radical scavenging assay	Above 200 µg/mL, the extract showed 80% scavenging activity	[39]
Antioxidant	Ethanol	Roots	DPPH free radical scavenging assay	Above 100 µg/mL, the extract showed 80% scavenging activity	[39]
Antioxidant	Methanol	Bark	Hydroxyl (^•^OH) radical scavenging assay	Exhibited activities with IC_50_ values of 3.6 mg/mL	[48]
Antioxidant	Methanol	Bark	Super oxide (SO) assay	Exhibited activities with IC_50_ values of 1.6 mg/mL	[48]
Antioxidant	Methanol	Bark	Nitric oxide (NO) radical scavenging assay	Exhibited activities with IC_50_ values of 3.6 mg/mL	[48]
Antioxidant	Methanol	Bark	Iron chelating property assay	Exhibited activities with IC_50_ values of 3.9 mg/mL	[48]
Antioxidant	Methanol	Bark	DPPH free radical scavenging assay	Exhibited activities with IC_50_ values of 0.7 mg/mL	[48]
Antioxidant	Methanol	Bark	2,2´-azinobis-3-ethylbenzothiazoline-6-sulfonic acid (ABTS) radical scavenging assays	Exhibited activities with IC_50_ values of 4.1 mg/mL	[48]
Antioxidant	Methanol	Leaves	DPPH free radical scavenging assay	Exhibited activities with EC_50_ value of 2.8 mg/mL	[57]
Antiplasmodial	Dichloromethane	Bark	Plasmodium falciparum Plasmodium falciparum lactate dehydrogenase assay	Extract exhibited activities with IC_50_ value of 5.1 µg/mL	[48]
Anti-protozoan	Aqueous	Bark	Serial two-fold dilution	Extract exhibited activities with MIC value of 9.7 mg/mL against *Trichomonas vaginalis*	[66]
Anti-pyretic	Methanol	Bark	In vivo experiments using female and male Sprague-Dawley rats	Extracts exhibited potential to reduce pyrexia in the induced rats and activities were time and concentration dependent with extracts showing activity as early as from 30 minutes and even at the lowest concentration of 100 mg/kg	[48]
Hypoglycaemic	Acetone	Stem bark	In vitro anti-diabetic and toxicity screening against murine C2C12 myoblasts, Chang liver cells and 3T3-L1 preadipocytes	Extracts had potential of 138.6% to lower blood glucose levels at a concentration of 50 µg/mL against 3T3-L1 preadipocytes and 100% against both C2C12 myoblasts and Chang liver cells.	[67]
Hypoglycaemic	Acetone	Stem bark	α-amylase inhibiting activity	Extract exhibited activity with IC_50_ value of 1.1 µg/mL	[67]
Hypoglycaemic	Acetone	Stem bark	α-glucosidase inhibiting activity	Extract exhibited activity with IC_50_ value of 50.6 µg/mL	[67]
Larvicidal	Dichloromethane	Bark	Larvicidal assay on *Culex quinquefascitus* larvae	Extracts exhibited activities with 60% mortality and IC_50_ value of 18.2 µg/mL	[48]
Larvicidal	Methanol	Bark	Larvicidal assay on *Culex quinquefascitus* larvae	Extracts exhibited activities with 47% mortality and IC_50_ value of 9.8 µg/mL	[48]
Cytotoxicity	Ethanol	Stem bark	3-(4,5-dimethylthiazol-2-yl)-2,5-diphenyl tetrazolium bromide (MTT) calorimetric assay	Extracts exhibited activities at 12.5 µg/mL showing 90% and 40% of viable 3T3-L1 preadipocytes and Chang liver cells, respectively of the control	[67]
Cytotoxicity	Ethanol	Stem bark	XTT (sodium 3’-[1-(phenyl amino-carbonyl)-3,4-tetrazolium]-bis-[4-methoxy-6-nitro] benzene sulfonic acid hydrate) colorimetric assay	Extracts exhibited activities with IC_50_ values >100.0 µg/mL in both Vero cells and MCF-7 cell line	[53]
Cytotoxicity	Methanol	Stem bark	3-(4,5-dimethylthiazol-2-yl)-2,5-diphenyl tetrazolium bromide (MTT) assay	Extracts exhibited activities with half maximal cytotoxic concentration (CC_50_) value of 3.7 mg/mL	[62]
Cytotoxicity	Dichloromethane	Bark	MTT cell proliferation assay	Extracts exhibited activities with the median lethal concentration (LC_50_) value of 512.0 µg/mL and 394.0 µg/mL against human embryonic kidney (HEK293) and human hepatocellular carcinoma (HepG2) cells, respectively	[48]
Cytotoxicity	Methanol	Bark	3-(4,5-dimethylthiazol-2-yl)-2,5-diphenyl tetrazolium bromide (MTT) calorimetric assay	Extract exhibited activities with CC_50_ value of 200.0 µg/mL and selective index (SI) value of 57.1	[23]
Cytotoxicity	Aqueous	Bark	MTT cell proliferation assay	Extracts exhibited activities in all the three human tumour cancer cell lines	[63]
Cytotoxicity	70% acetone	Stem bark	Cytotoxicity assay on MT2 cells	Extracts showed cell death of 22.7% after 36 h at the highest concentration tested of 15 µg/mL	[50]
Cytotoxicity	Chloroform	Stem bark	Cytotoxicity assay on MT2 cells	Extracts showed cell death of 27.6% after 36 h at the highest concentration tested of 15 µg/mL	[50]
Cytotoxicity	Ethyl acetate	Stem bark	Cytotoxicity assay on MT2 cells	Extracts showed cell death of 17.1% after 36 h at the highest concentration tested of 15 µg/mL	[50]
Antimutagenicity	Methanol	Leaves	Ames test	Extract exhibited weak antimutagenic activities with 23.2% inhibition of 4-nitroquinoline 1-oxide in *Salmonella typhimurium* TA98 and 21.3% in strain TA100 at the assayed concentration of 5 mg/mL	[57]
